# Microbial Egress: A Hitchhiker's Guide to Freedom

**DOI:** 10.1371/journal.ppat.1004201

**Published:** 2014-07-24

**Authors:** Ana Traven, Thomas Naderer

**Affiliations:** Department of Biochemistry and Molecular Biology, Monash University, Clayton, Victoria, Australia; The University of North Carolina at Chapel Hill, United States of America

## The Restaurant at the End of the Infection—Macrophages as Host Cells

**Figure 1:**
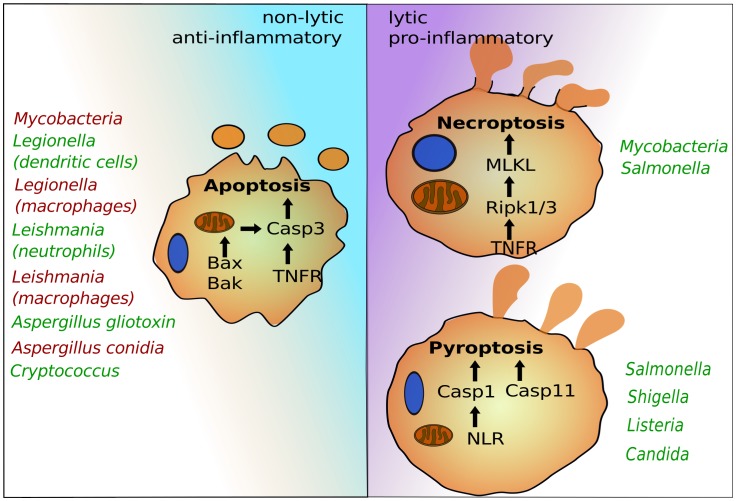
The programmed cell death pathways apoptosis, pyroptosis, and necroptosis play critical roles in egress of intracellular pathogens. Intrinsic apoptosis is dependent on mitochondrial damage by Bax and Bak and activation of caspase-3. Caspase-3 can also be activated via cell surface receptors such as TNFR in the pathway of extrinsic apoptosis. Pyroptosis depends on caspase-1, which is activated by cytosolic NLRs, and caspase-11, for which the precise signaling pathway needs to be determined. Necroptosis requires Ripk1 and 3 and MLKL. Representative intracellular pathogens are listed that modulate these host suicide programs, either by blocking (red) or inducing (green) their activation.

After colonization or invasion, intracellular pathogens seek host cells to establish infections and facilitate replication. This lifestyle provides essential nutrients and protection from immune systems. Several host cells—in particular professional phagocytes, such as monocytes, macrophages, and neutrophils—have developed antimicrobial mechanisms to deplete the replicative niche of these intracellular microbes. Programmed cell death pathways are frontline defense mechanisms, whereby host cell suicide blocks intracellular microbial replication and exposes them for immune attack [1]. Recent advances have uncovered several programmed cell death pathways that are highly regulated to ensure proper immune responses. Besides canonical apoptosis, which initially preserves host cell integrity and is largely anti-inflammatory, macrophages can induce pyroptosis and necroptosis, which are both highly lytic and trigger proinflammatory signals (Figure 1). Pyroptosis depends on the cysteine-aspartic proteases, caspase-1 or caspase-11, which are activated by cytosolic pattern recognition receptors [1], [2]. Necroptosis is regulated by the TNFR (tumor necrosis factor receptor)-associated kinases, Ripk1 and 3, and is executed by MLKL (mixed lineage kinase domain-like) [3]. Successful intracellular pathogens must suppress programmed cell death signals during the replicative or latent phase but can potentially induce these signals to promote egress and dissemination. The timing and specificity of the cell death signal can dramatically influence pathogen or host survival, suggesting a complex interplay exists between inducing potent immunity and promoting pathogen egress. Most work in this area has focused on bacteria and parasites, but more recent studies have provided exciting evidence that fungal pathogens likewise modulate host cell death pathways.

## Life, Death, and Everything in between—Microbial Egress and Apoptosis

Generally, intracellular pathogens suppress apoptosis to promote replication. In addition, apoptosis can also block efficient escape, as is the case with *Mycobacterium tuberculosis* (Figure 1) [4]. Avirulent *Mycobacteria* that fail to inhibit apoptosis become trapped, as the plasma membrane remains intact for several hours after host cell death. This leads to uptake and digestion of the apoptotic cell together with *Mycobacteria* by uninfected macrophages [4]. To prevent this, *Mycobacteria* induce necroptosis, which results in plasma membrane rupture, bacterial release, and survival [5]. The ability to interfere with cell death signaling can depend on the host cell involved. For example, dendritic cells, but not macrophages, rapidly undergo apoptosis and prevent intracellular replication of *Legionella* [6]. Another example is the protozoan parasite *Leishmania*, which also blocks apoptosis in macrophages [7]. However, the first phagocytes encountered during infection are neutrophils. After phagocytosis by neutrophils, *Leishmania* manages to move into a permissive niche of macrophages by inducing apoptosis of the infected neutrophil [8]. This is partly because the “find-me/eat-me” signal displayed by the apoptotic infected neutrophil triggers uptake of parasites and anti-inflammatory responses of macrophages that enable intracellular parasite survival [9]. Fungal pathogens also target apoptosis. Intracellular *Aspergillus* conidia block macrophage apoptosis by up-regulating prosurvival signaling, which leads to inhibition of caspase-3 activity [10]. This is thought to provide immune protection for conidia inside the phagocytes. For egress, intracellular conidia generate germ tubes and hyphae that appear to pierce through host membranes [11]. Hyphae secrete gliotoxin, which triggers intrinsic apoptosis of host immune cells by inducing mitochondrial pore formation via Bak and activation of caspase-3 [12], [13], but its role during egress remains ill defined. The facultative intracellular fungus *Cryptococcus* induces host cell apoptosis, which, in contrast to *Mycobacteria*, may in this case promote escape from macrophages. Intracellular *Cryptococcus* produces large amounts of the polysaccharide-enriched capsule, which is frequently shed into discrete host vesicles [14]. At least two capsule glycans, galactoxylomannan (GalXM) and glucuronoxylomannan (GXM), have been shown to activate apoptosis in macrophages either by inducing cell surface death receptors or via intrinsic Bax/Bak-dependent apoptosis [15]–[18]. While the pathogenic fungus *Candida albicans* also modulates apoptotic signaling [19], there is little evidence so far that egress from macrophages relies on host cell apoptosis [20]–[22].

## Mostly Harmless—Pathogen Escape without Host Cell Lysis

Cytosolic microbes can also egress and spread between host cells via propulsive forces due to actin polymerization, which generates protrusions at the plasma membrane [23]. These protrusions are engulfed by neighboring cells, facilitating transfer of intracellular pathogens, such as *Shigella*, without host cell death. Vacuolar pathogens can also egress without host cell lysis. Inclusions of *Chlamydia* recruit and activate the myosin motor complex and are extruded from cells via actin polymerization [24]. *Cryptococcus* is known to hijack the ability of (phago)lysosomes to fuse directly with the plasma membrane, resulting in the release of lysosomal content including fungal cells [25], [26]. This form of nonlytic exocytosis or “vomocytosis” depends on the interplay between several host factors such as phagosomal pH, inflammatory signals, host cell microtubules, actin polymerization, and exocytosis signals [27], [28]. *C. albicans* has also been reported to escape from macrophages without concomitant macrophage lysis in a small fraction of cases (<1%) [29].

## So Long and Thanks for All the Inflammatory Cell Death

In contrast to nonlytic escape, pathogens can also induce extensive host cell damage and death during egress. While some bacteria can escape from their vacuoles without causing concomitant host cell death [30], cytosolic bacteria, as well as microbial molecules that reach the cytosol, can be detected by NOD-like receptors (NLRs) that induce caspase-1-dependent pyroptosis (Figure 1) [1]. In addition, contamination of the cytosol by lipopolysaccharide (LPS) triggers pyroptosis via the activation of caspase-11 [31]. Others, like *Shigella*, induce caspase-1-dependent pyroptosis by triggering phagosome rupture caused by secretion of ion channels [32]. Vacuolar pathogens can also induce pyroptosis by secreting effector proteins that are recognized by NLRs into host cytosols [1], [33]. Besides pyroptosis, intracellular *Salmonella* may also activate necroptosis for escape from macrophages, at least under certain conditions [34]. Both pyroptosis and necroptosis trigger potent antimicrobial immune responses, but these types of lytic host cell death also result in rapid release of intracellular pathogens, enabling dissemination and replication in rich extracellular niches [1], [34]. Very recently, intracellular *C. albicans* has been shown to trigger caspase-1-dependent pyroptosis in macrophages, the first evidence of a fungal pathogen causing pyroptosis [20], [35]. This new discovery questions the long-standing view that *C. albicans* hyphal filaments kill macrophages by piercing host membranes [36]. Instead, phenotypes of several *Candida* mutants suggest that filaments contain additional features likely related to cell surface characteristics, which are necessary for induction of pyroptosis [20], [37], [38]. The cell wall carbohydrates, including β-glucans, are highly immunogenic because they are sensed by several macrophage lectin receptors, such as Dectin-1, that enable priming of the NLRP3 inflammasome [39], [40]. There is, however, little evidence that the β-glucans can trigger pyroptosis directly, suggesting that *Candida* may depend on additional factors. Other fungal pathogens, such as *Aspergillus fumigatus*, also induce caspase-1 activation [41]. Thus, pyroptosis could be a common mechanism for fungal egress. Of note, *C. albicans* hyphae can also egress from macrophage in a pyroptosis-independent manner, particularly when fungal loads are high [20], [35]. The precise nature of the pyroptosis-independent killing of macrophages by *C. albicans* remains to be determined, but it was proposed that it could represent necrotic death when macrophages are overloaded with fungal cells [35].

## And Another Thing: Egress Pathways Determine Clinical Outcome

Induction of programmed cell pathways can dramatically determine the health of the host and the pathogen, suggesting that microbial egress via host cell suicide needs to be carefully balanced. For instance, caspase-1-dependent pyroptosis triggers potent antimicrobial responses targeted at escaped *Salmonella* [1], while caspase-11-dependent pyroptosis (in the absence of caspase-1 activation) actually promotes extracellular bacterial replication and dissemination to the detriment of the host [42]. Similarly, caspase-1-dependent immune response can trigger antifungal inflammation [40], but “hijacking” macrophage pyroptosis promotes rapid egress of *C. albicans* from macrophages [20], [35]. Not all proinflammatory responses result in clearance of intracellular pathogens. For example, *Salmonella* can utilize type 1 interferon signaling for egress from macrophages and for survival in mice [34], [42]. Furthermore, some microbes thrive under proinflammatory conditions, including *Listeria* and some *Leishmania* species, suggesting that the timing and manner of programmed cell death is critical for pathogen egress and disease [43]–[45]. In the case of tuberculosis, the level of inflammatory responses determines macrophage cell death signaling, disease progression, and pathogen clearance [5]. Finally, pathogen-provoked host cell death itself can contribute to disease outcome, as LPS-induced septic shock results from uncontrolled caspase-11-dependent pyroptosis [2]. Similarly, while caspase-1-dependent secretion of the proinflammatory cytokines interleukin-1β (IL-1β) and interleukin-18 (IL-18) has potent antifungal properties, symptoms of vaginal candidiasis are largely caused by sustained influx of neutrophils due to IL-1β secretion [46]. Given that immunopathology is also associated with fatal candidiasis [47], the fungal egress pathway in macrophages may directly contribute to disease progression.
